# Biotransformation of common NSAIDs by *Cunninghamella* species and resulting metabolite toxicity

**DOI:** 10.1038/s41598-026-58519-6

**Published:** 2026-06-18

**Authors:** Aleksandra Pietrzak, Barbara Dąbrówka, Justyna Popiół-Adamska, Agnieszka Gunia-Krzyżak, Dorota Żelaszczyk, Paulina Koczurkiewicz-Adamczyk, Kamil Piska, Paweł Żmudzki, Elżbieta Pękala, Karolina Słoczyńska

**Affiliations:** 1 Department of Pharmaceutical Biochemistry, Faculty of Pharmacy, Jagiellonian University Medical College, Medyczna 9 St., Kraków, 30-688 Poland; 2 Doctoral School of Medical and Health Sciences, Jagiellonian University Medical College, Łazarza 9 St., Kraków, 31-530 Poland; 3 Department of Experimental Dermatology and Cosmetology, Faculty of Pharmacy, Jagiellonian University Medical College, Medyczna 9 St., Kraków, 30-688 Poland; 4 Department of Medicinal Chemistry, Faculty of Pharmacy, Jagiellonian University Medical College, Medyczna 9 St., Krakow, 30-688 Poland; 5 Small Molecule Analysis Laboratory, Center for the Development of Therapies for Civilization and Age-Related Diseases, Jagiellonian University Medical College, Medyczna 9 St., Kraków, 30-688 Poland

**Keywords:** Biotransformation, *Cunninghamella*, NSAIDs, Toxicity, Biochemistry, Biotechnology, Drug discovery

## Abstract

Nonsteroidal anti-inflammatory drugs (NSAIDs) such as diclofenac (DCF), ibuprofen (IBU), and ketoprofen (KET) are commonly found in the environment and pose potential toxicological risks. This study investigates the microbial biotransformation of these NSAIDs by *Cunninghamella* spp. and evaluates the toxicity of the *Cunninghamella*-processed samples. The results demonstrate that *Cunninghamella* efficiently biotransformed DCF and IBU into multiple metabolites, whereas the biotransformation of KET was negligible. Microbial treatment reduced the ecotoxicity of IBU and DCF, as confirmed by Microtox^®^ and algal growth inhibition assays. *In vitro* cytotoxicity tests using human hepatocytes (HepG2), neuroblastoma cells (SH-SY5Y), astrocytes, and rat cardiomyocytes (H9c2) showed lower toxicity of the biotransformation products compared to the parent compounds. The Ames assay revealed no mutagenicity for any of the samples. Endocrine disruption assays indicated a loss of antagonistic estrogenic and androgenic effects post-biotransformation, with minor estrogenic agonistic activity observed. In conclusion, fungal biotransformation by *Cunninghamella* appears to be a promising strategy for reducing the environmental toxicity of NSAIDs. However, the generation of transformation products with altered biological activity underscores the importance of combining analytical identification with comprehensive toxicological assessment.

**Supplementary Information:**

The online version contains supplementary material available at https://doi.org/10.1038/s41598-026-58519-6.

## Introduction

Nonsteroidal anti-inflammatory drugs (NSAIDs) are among the most commonly used drugs worldwide. More than 30 million people take NSAIDs daily, as they are often available over-the-counter^1^. Ibuprofen (IBU), diclofenac (DCF), and ketoprofen (KET) are the most frequently used NSAIDs^2–4^.

In recent years, the environmental ubiquity of DCF, IBU, and KET, along with their potential detrimental effects on aquatic ecosystems, has raised concerns. Therefore, these compounds are considered emerging organic contaminants (EOCs)^5,6^. The lowest reported environmental concentration of DCF was below 1 ng/L in drinking water, whereas the highest concentration in wastewater influent reached 836 µg/L^7,8^. IBU concentrations range from 3 ng/L in groundwater to 1673 µg/L in wastewater^5,7^, while KET has been detected in concentrations ranging from 0.16 ng/L in groundwater to 260 µg/L in wastewater influent^6^. Even at low concentrations, these agents can induce numerous harmful ecotoxicological effects in aquatic organisms, affecting, among others, growth, development, reproduction, immune response, and survival^5,6^. NSAIDs can enter aquatic ecosystems via wastewater discharge, as conventional wastewater treatment systems struggle to completely eliminate these compounds^5^.

Mycoremediation, defined as the use of fungi and their enzymatic systems to degrade, transform, or remove environmental contaminants, has emerged as a promising approach for mitigating pharmaceutical pollution in the environment^9^. Previous studies have reported fungal-mediated biotransformation of pharmaceuticals with high removal efficiency and the formation of non-toxic products^10–16^. Fungi of the genus *Cunninghamella* are naturally occurring, non-pathogenic soil microorganisms. Previous studies have shown that *Cunninghamella* spp. are capable of degrading a wide range of pollutants, including, among others, sunscreen agents, drugs, fungicides, insecticides, and industrial chemicals^12,17–22^. The versatility in degrading various xenobiotics makes these microorganisms potentially useful in bioremediation. While biological treatment processes currently employed in wastewater treatment plants rely predominantly on bacterial consortia, increasing evidence suggests that these systems are often insufficient for the effective removal of some pharmaceutical residues. Consequently, there is a growing need to explore alternative or complementary microbial systems capable of degrading xenobiotics. Filamentous fungi such as *Cunninghamella* spp. represent an attractive alternative due to their remarkable physiological robustness, including tolerance to temperature fluctuations, high concentrations of chemical contaminants, and a broad pH range spanning from strongly acidic to alkaline conditions. Moreover, their rapid biomass production, long life cycle, low nutritional requirements, and the formation of extensive mycelial networks enhance pollutant adsorption and transformation^10–12,23^.

Previous papers on the biotransformation of DCF, IBU, and KET by fungal species have primarily focused on the transformation products (TPs) generated and/or the efficiency of the process, without addressing the safety of these products. Marco-Urrea et al. examined the ability of *Trametes versicolor* to remove DCF^13^ and IBU^24^, and further assessed the ecotoxicity of the fungus-treated medium. Alharbi et al.^11^ studied the degradation of DCF by *T. versicolor* laccase. The toxicity of the solution after enzymatic treatment was assessed using a bacterial luminescence toxicity assay. To our knowledge, no investigation has yet been published on the biotransformation of KET by *Cunninghamella* spp. and *Aspergillus niger*. Moreover, studies on the safety of obtained TPs are scarce and focus solely on the ecotoxicological endpoints, neglecting the assessment of remaining risks. Considering the widespread occurrence of both fungi and NSAIDs in the environment, fungal biotransformation of these contaminants is highly probable under environmental conditions. Importantly, environmental exposure typically involves complex mixtures of NSAIDs, their metabolites, and degradation products, whose combined effects may alter overall toxicity through synergistic or antagonistic interactions. Consequently, increasing attention is being paid to the study of complex environmental samples rather than individual substances.

Taking these considerations into account, the main objectives of this study were to: (i) assess the ability of *Cunninghamella* strains to remove DCF, IBU, and KET; (ii) comprehensively evaluate the toxicological implications of fungal biotransformation, including changes in ecotoxic, cytotoxic, mutagenic, and endocrine-disrupting properties of the resulting products; (iii) comparatively assess the biotransformation potential of *Cunninghamella* against fungi with well-documented biodegradation capabilities; (iv) distinguish between fungal cytochrome P450 (CYP)-mediated and non-CYP-mediated biotransformation pathways; and (v) characterize and compare the identified biotransformation products with predicted mammalian metabolites. By combining a comparative biodegradation analysis with the elucidation of TP structures and their potential toxicological consequences, this study addresses a significant gap in the current literature.

## Results and discussion

### Microbial biotransformation

The ability of *Cunninghamella* strains to utilize IBU and DCF was observed (Table 1). After 7-day incubation, all *Cunninghamella* strains showed complete IBU removal. For *A. niger* and *T. versicolor*, 73% and 90% of the initial IBU concentration, respectively, remained in the flask. Conversely, Marco-Urrea et al.^24^ found that IBU was degraded by *T. versicolor* to non-detectable levels after 7 days of treatment. Interestingly, Kasonga et al.^25^ reported that the IBU removal efficiency by *A. niger* approached 100% on the 14th day of incubation.

**Table 1:** Parent compounds residues after biotransformation by tested fungal strains. Data are presented as the mean value (*n* = 6).

Tested compound/fungal strain	Parent compound residue (%)				
	*C. echinulata*	*C. blakesleeana*	*C. elegans*	*A. niger*	*T. versicolor*
DCF	15	25	9	82	14
IBU	0	0	0	73	90
KET	100	100	99	98	42

Among the tested *Cunninghamella* strains, DCF was most efficiently removed by *Cunninghamella elegans*. The observed degrading ability of *C. elegans* is comparable to the values reported previously^26^. As for *Cunninghamella blakesleeana*, 75% of DCF was eliminated during incubation. Meanwhile, the biodegradation efficiency of *Cunninghamella echinulata* was similar to that of *T. versicolor*. These results are in line with Yang et al.^27^, who reported that *T. versicolor* treatment resulted in about 90% elimination of DCF. Osorio-Lozada et al.^26^ demonstrated that *C. echinulata* biotransformed 100% of DCF within 5 days. Almost complete removal of DCF occurred also with *T. versicolor* pellets^13^. In contrast, Ercoli et al.^28^ revealed that the removal rate by *T. versicolor* reached only 63%. In the present study, *A. niger* showed very low DCF removal capacity. This was not consistent with previous reports, which demonstrated that *A. niger* removed 74% of the initial DCF concentration^29^. Aracagök et al.^10^ found that after 48 h of incubation *A. niger* showed a 100% DCF removal rate. Additionally, Kasonga et al.^25^ reported that *A. niger* removed 91% of DCF by day 7.

Regarding KET, among the tested fungal strains, only *T. versicolor* was able to partially remove this compound. Marco-Urrea et al.^23^ showed that *T. versicolor* eliminated KET to non-detectable levels within 24 h when it was added at 10 mg/L, whereas at 40 µg/L it was almost completely removed after 5 h.

It can be concluded that IBU was the most easily removed compound by all tested *Cunninghamella* fungi, and DCF elimination by *C. elegans* also gave satisfactory results. The observed differences in the biotransformation efficiency of the tested NSAIDs can presumably be explained by the molecular structure and physicochemical properties of these drugs. Ibuprofen, with its simple aliphatic moiety and readily accessible positions for hydroxylation, provides suitable substrates for fungal enzymes, which promote effective oxidation. In the case of diclofenac, aromatic rings and chloroaniline groups allow oxidation and conjugation, albeit with lower efficiency. Conversely, ketoprofen, containing a benzophenone moiety and a benzoyl group that strongly stabilizes the aromatic core, shows low susceptibility to hydroxylation initiation, resulting in no noticeable biotransformation under experimental conditions. Furthermore, the pKa and ionization of ketoprofen at the culture pH (pH = 7.05) may limit its penetration into the biomass, reducing the bioavailability of the substrate^6,30^.

The existing discrepancy between our results and those reported by other authors may be attributed to the methodology used. Many authors used much lower initial drugs concentrations than in our study^10,24^, along with a lower mass of fungal pellets^23^. Moreover, some authors investigated the simultaneous removal of several compounds or conducted experiments over a relatively long period of time^25^. Ercoli et al.^28^ concluded that the removal rate of DCF varies depending on the microorganism. The effect of incubation time on fungal enzymatic activity is substantial, influencing the production and stability of crude enzymes^25^.

It should also be acknowledged that the present study did not include a heat-inactivated fungal control. Therefore, the contribution of abiotic adsorption of pharmaceuticals onto fungal biomass cannot be fully excluded, and part of the observed decrease in substrate concentration may have resulted from adsorption processes in addition to metabolic conversion. This represents a limitation of the study and should be considered when interpreting the overall removal efficiency. Nevertheless, such a mechanism may still be considered environmentally relevant, as the reduced bioavailability of the parent compound could contribute to the mitigation of its potential toxic effects.

### DCF biodegradation products

A total of 11 TPs were detected after DCF incubation with the tested fungi, and the proposed structures were developed based on the obtained accurate *m/z* values in positive and/or negative ionization, obtained fragmentation spectra and biochemical logic (Figs. S1, S2, S3, S4, Table 2). The mass spectra of all identified derivatives are provided in the Supplementary Material (Figs. S5–S15). In the case of three TPs exhibiting the same major ion at *m/z* 312 [M + H]^+^, a mass increase of 16 was observed relative to the parent compound, indicating monohydroxylation of DCF moieties. Based on the literature, these hydroxylated TPs are most likely 4’-hydroxydiclofenac (DCF-1), 3’-hydroxydiclofenac (DCF-2), and 5-hydroxydiclofenac (DCF-3), and have been identified in human plasma and urine. Notably, both TPs with hydroxylation on the benzene ring containing the two chlorine atoms were observed in all fungal cultures, whereas the intermediate hydroxylated on the non-chloride ring was identified solely in *T. versicolor*. The fragment ions of DCF-1 and DCF-2 correspond to CO_2_ loss and two subsequent losses of HCl^11,26,31,32^. Fragmentation of DCF-3 shows characteristic multiple losses of CO and Cl, and the additional fragment at *m/z* 166 (i.e., 2-(2-amino-4-hydroxyphenyl)acetic acid) provides strong evidence that the hydroxylation occurred on the non-chlorinated ring^31–33^.

**Table 2:** Hypothesized structures, molecular formulas, and selected parameters of DCF biotransformation products obtained in the tested fungal strains (*C*. *echinulata − 1*, *C. blakesleeana – 2*,* C. elegans – 3*,* A. niger – 4*,* T. versicolor – 5).*

Peak ID	Chemical structure	Formula	*R*_t_ (min)	[M + H]^+^/ [M + H]^−^	Major daughter ions	MetaSite prediction	Proposed biotransformation reaction	Fungal strain	References
DCF		C_14_H_11_Cl_2_NO_2_	7.33	296	250, 215	–	–	–	^31,72,73^
DCF-1		C_14_H_11_Cl_2_NO_3_	5.97	312	267, 230, 196	+	Aromatic hydroxylation	1,2,3,4,5	^11,26,31,32,73^
DCF-2		C_14_H_11_Cl_2_NO_3_	6.07	312	267, 230, 196	+	Aromatic hydroxylation	1,2,3,4,5	^73^
DCF-3		C_14_H_11_Cl_2_NO_3_	7.61	312	266, 230, 194, 166	+	Aromatic hydroxylation	5	^26,31–33,73^
DCF-4		C_14_H_12_ClNO_3_	5.50	276	258, 232, 196	+	Oxidative dehalogenation	1	^33^
DCF-5		C_14_H_10_ClNO_2_	5.74	259	224, 180	–	Aromatic hydroxylation, intramolecular amidation and dehalogentaion	1	^31,32^
DCF-6		C_14_H_12_ClNO_2_	5.82	262	nd	+	Reductive dehalogenation	1	–
DCF-7		C_14_H_9_Cl_2_NO_3_	6.26	310	291, 263, 229, 194	–	Aromatic hydroxylation and dehydrogenation	1	^31,32,73^
DCF-8		C_17_H_16_Cl_2_N_2_O_5_S	7.10	432	341, 331, 296, 261	–	Cysteine conjugation of hydroxylated DCF	1,2,3	^75^
DCF-9		C_16_H_14_Cl_2_N_2_O_3_	5.15	352	nd	–	Glycine conjugation	3	^35^
DCF-10		C_14_H_10_Cl_2_NO	7.88	278	243, 215, 214, 208, 180, 171	–	Intramolecular amidation	4	^31^
DCF-11		C_13_H_9_Cl_2_NO_2_	7.45	282	246, 212	–	Aromatic hydroxylation, dehydrogenation, reduction of the carboxylic acid moiety	5	^32^

TPs DCF-4, DCF-5, DCF-6, and DCF-7 were detected only in *C. echinulata*. Based on our data and literature analysis^33^, it can be speculated that product DCF-4 is 2-(2-((2-chloro-6-hydroxyphenyl)amino)phenyl)acetic acid. DCF-5 showed a molecular ion at *m/z* 259. Based on its fragmentation pattern analysis, it can be assumed that the compound is 1-(2-chloro-4-hydroxyphenyl)indolin-2-one^31,32^. The mass spectrum of DCF-6 exhibited a molecular ion peak [M + H]^+^ at *m/z* 262, likely corresponding to 2-(2-((2-chlorophenyl)amino)phenyl)acetic acid. The structure of this compound was predicted using MetaSite. The molecular ion peak [M + H]^+^ at *m/z* 310 and the fragmentation pattern of DCF-7 were consistent with (*E*)-2-(6-((2,6-dichlorophenyl)imino)-3-oxocyclohexa-1,4-dien-1-yl)acetic acid, which is thought to be formed via oxidation of DCF-3 by dehydrogenation^32^.

The mass spectrum of DCF-8 demonstrated mass transitions supporting the identification of a cysteine conjugate of hydroxylated DCF, previously reported in *C. echinulata*^34^. This compound was identified in all *Cunninghamella* strains, whereas the probable glycine conjugate (DCF-9), was produced solely by *C. elegans*. This product was identified* in vitro* in primary human hepatocytes^35^.

Based on the precursor *m/z* and MS fragmentation pattern, the structure of DCF-10 was proposed as 1-(2,6-dichlorophenyl)indolin-2-one^31^. This compound was formed exclusively by *A. niger* through intramolecular amidation of the carboxylic acid with the primary amine. Not only were hydroxy derivatives generated by *T. versicolor*, but also DCF-11, which was proposed to be (*E*)-4-((2,6-dichlorophenyl)imino)-3-(hydroxymethyl)cyclohexa-2,5-dien-1-one. Its characteristics fragment ions indicate two subsequent losses of HCl^32^.

Our results are in agreement with those obtained by previous authors^13^, who identified two TPs formed via hydroxylation at the *para* position of the amine group on the benzene rings in biotransformation experiments with *T. versicolor* pellets and *T. versicolor* laccase. In our study, however, two additional *T. versicolor* TPs, DCF-2 and DCF-11, were detected. Ibrahim et al.^36^ revealed that *A. niger* NRRL 2295 converted DCF to 4’-hydroxydiclofenac, 5-hydroxydiclofenac, and 4’-hydroxydiclofenac lactam, whereas *A. niger* NRRL 599 (used in our study) transformed this compound only to 4’-hydroxydiclofenac. Similarly, in our experiment, this strain produced 4’-hydroxydiclofenac; however, we also detected 3’-hydroxydiclofenac and DCF lactam.

A study by Quinn et al.^37^ showed that *C. elegans*, both in alginate-immobilized and planktonic cultures, was capable of producing 4’-hydroxydiclofenac as the main product and 3,4’-dihydroxydiclofenac in smaller amounts. Ibrahim et al.^36^ reported that *C. echinulata* generated 4’-hydroxydiclofenac, *C. elegans* produced both 4’-hydroxydiclofenac and its lactam, whereas *C. blakesleeana* produced 4’-hydroxydiclofenac and 5-hydroxydiclofenac. In our experiments, *C. echinulata* generated six additional TPs, while *C. elegans* produced also 3’-hydroxydiclofenac and two phase II products, however, without the lactam and dihydroxy derivative. *C. blakesleeana* generated 3’-hydroxydiclofenac instead of 5-hydroxydiclofenac and a cysteine conjugate of hydroxylated DCF. Rydevik et al.^34^ reported the formation of glutathione and cysteine conjugates by *C. elegans*. In our study, we observed the formation of cysteine conjugates by all *Cunninghamella* strains.

When *C. echinulata* was incubated with the CYP450 inhibitor ABT, the fungal degradation ability of DCF was found to be negligible (Fig. S16), confirming the role of the CYP450 enzymatic system in DCF degradation. DCF 4’-hydroxylation is primarily catalyzed by CYP2C9, with a contribution from CYP3A4.

*In silico* metabolism predictions for DCF did not fully reflect the in vitro observations, as only five in vitro TPs were identified by MetaSite (Fig. S17). Regarding the conjugation products, the tool version 6.0.1 predicts metabolic transformations related only to phase I reactions^38^. This limitation reduces its relevance for the comprehensive exploration of compound metabolism. The top four most likely metabolites were DCF-1, DCF-3, 3-hydroxydiclofenac, and 2-hydroxydiclofenac. However, the latter two metabolites were not observed in vitro, and the overlap between experimentally detected and predicted metabolites is summarized in a Venn diagram (Fig. S18). Thus, software predictions should always be compared with *in vitro* or *in vivo* results to identify true-positive metabolites. Nevertheless, the *in silico* metabolite prediction method significantly accelerates the generation of possible metabolite lists and also assists in identifying previously unreported metabolic reactions (e.g., DCF-6). It should be also highlighted that MetaSite is a computational tool that predicts metabolic transformations related to CYP450 enzymes and FMO-mediated reactions in phase I metabolism, whereas the diversity of fungal species and enzymes enables a much broader potential for metabolic variation, which may explain the observed discrepancies^39^.

### IBU biodegradation products

Five IBU derivatives were identified in fungal post-culture extracts (Figs. S19, S20, Table 3). The mass spectra of all identified IBU derivatives are provided in the Supplementary Material (Figs. S21–S25). Notably, as IBU and its TPs are all carboxylic acids, MS studies of its biotransformation are usually conducted in negative ion mode. Three peaks exhibited identical *m/z* of 221 and most likely resulted from the oxidation of the parent compound’s isopropyl chain, yielding 1-hydroxyibuprofen (IBU-1), 2-hydroxyibuprofen (IBU-2), and 3-hydroxyibuprofen (IBU-3). Both IBU-1 and IBU-2 fragmented with an intense product ion signal at *m/z* 177, indicating the loss of CO_2_. In the case of IBU-1, this was followed by the loss of H_2_O. All three hydroxy derivatives of IBU are known human metabolites and were produced by both *C. blakesleeana* and *C. elegans*. In *C. echinulata* samples, only 2- and 3-hydroxyibuprofen were detected. Quinn et al.^37^, in studies with *C. elegans*, reported the formation of hydroxylated IBU derivatives and carboxyibuprofen in planktonic cells, whereas only hydroxylated metabolites were detected in alginate-immobilised cultures.

**Table 3:** Hypothesized structures, molecular formulas, and selected parameters of IBU biotransformation products obtained in the tested fungal strains (*C. echinulata − 1*, *C. blakesleeana – 2*,* C. elegans – 3*,* A. niger – 4*,* T. versicolor – 5).*

Peak ID	Chemical structure	Formula	*R*_t_ (min)	[M + H]^+^/ [M + H]^−^	Major daughter ions	MetaSite prediction	Proposed biotransformation reaction	Fungal strain	References
IBU		C_13_H_18_O_2_	7.59	205	161, 119, 91	–	–	–	^25,75,76^
IBU-1		C_13_H_18_O_3_	4.42	221	204, 177, 159	+	Aliphatic hydroxylation	2,3,4	^76,77^
IBU-2		C_13_H_18_O_3_	5.25	221	443, 177, 133, 119, 91	+	Aliphatic hydroxylation	1,2,3,5	^76–78^
IBU-3		C_13_H_18_O_3_	5.53	221	236, 159	+	Aliphatic hydroxylation	1,2,3	^77,78^
IBU-4		C_13_H_18_O_4_	9.50	237	193	–	Double aliphatic hydroxylation	5	^24,76,77^
IBU-5		C_19_H_26_O_9_	4.98	397	221, 177, 175, 113	–	Glucuronide conjugation of hydroxylated IBU	3	^77,79^

1-Hydroxyibuprofen was the only TP observed during incubation with *A. niger*, whereas *T. versicolor* produced 2-hydroxyibuprofen and 1,2-dihydroxyibuprofen. The latter TP was characterized by a mass transition realted to decarboxylation. Marco-Urrea et al.^24^ reported the formation of 1-hydroxyibuprofen and 2-hydroxyibuprofen during IBU degradation by *T. versicolor*. These by-products were subsequently degraded to 1,2-dihydroxyibuprofen. Analysis of our *T. versicolor* post-culture samples indicated the presence of 2-hydroxyibuprofen and 1,2-dihydroxyibuprofen, but not of 1-hydroxyibuprofen. Hutt et al.^40^ revealed that *C. elegans* catalyzed the oxidation of IBU to its 2-hydroxy derivative, whereas *C. echinulata* and *A. niger* were not able to biotransform the drug. *C. elegans* also carried out a conjugation reaction with IBU. The hydroxylated form of IBU was likely conjugated to glucuronic acid, yielding IBU-5. The transition from *m*/*z* 397 to 221 is consistent with the loss of a glucuronide moiety.

The involvement of CYP450 enzymes in the degradation of IBU by *C. elegans* has been confirmed through inhibition studies. The inclusion of ABT in the cultures prevented the biotransformation of the drug. In humans, CYP2C9 has been identified as the primary enzyme responsible for the formation of all the oxidative metabolites of IBU (Fig. S26).

Among the five IBU fungal TPs detected in the experiment, MetaSite predictions matched well with three metabolites, but failed to identify 1,2-dihydroxyibuprofen and hydroxyibuprofen glucuronide. The software assigned the highest likelihood of formation to IBU-2 and 2-(4-(2-methylprop-1-en-1-yl)phenyl)propanoic acid, the latter not found in our experimental samples. Two other *in silico* metabolites were IBU-1 and an aliphatic carbonylation product, which was also not observed in vitro (Fig. S27). The discrepancies observed between the in vitro and *in silico* results may, similarly to the case of DCF, be attributed to the limited range of enzymes incorporated into the MetaSite prediction model, as illustrated by the Venn diagram comparing predicted and experimentally detected metabolites (Fig. S18).

### KET biodegradation products

Four KET TPs were detected across the tested fungal systems (Figs. S28, S29, Table S1) and their mass spectra are provided in the Supplementary Material (Figs. S30–S33). The new peaks corresponding to KET-1, KET-2, and KET-3 exhibited identical product ion spectra and were suspected to be hydroxylation products at three different sites of the parent compound. The exact chemical structure of KET-1 was not confirmed; however, as a hydroxy derivative of the parent compound, it likely corresponds to 2-(3-benzoylphenyl)-3-hydroxypropanoic acid, predicted by MetaSite. This compound was produced solely by *C. elegans.* KET-2 likely corresponds to 2-(3-(4-hydroxybenzoyl)phenyl)propanoic acid. Its fragmentation yielded a major daughter ion at *m/z* 225 [M-CO_2_]^−^, supporting a reliable identification^23,41^. The molecular ion peak of KET-3 and fragment ions are consistent with those of 2-(3-(3-hydroxybenzoyl)phenyl)propanoic acid^41^. The biotransformation pathway for KET-4 involved reduction of the ketone group, a common metabolic pathway reported previously in several species^42^. Fragmentation pattern analysis confirmed the loss of CO_2_^23,41^. KET-4 was the only TP formed by *A. niger*, whereas *T. versicolor* produced KET-2, KET-3, and KET-4. Marco-Urrea et al.^23^ previously reported the production of KET-2, and KET-4 by *T. versicolor*. Moreover, the authors identified for the first time in biological systems 2-(3-benzoyl-4-hydroxyphenyl)-propanoic acid. KET TPs formed by hydroxylation of the aromatic ring (KET-2 and KET-3) and by reduction of the ketone group (KET-4) were also identified in human urine^41^.

MetaSite predicted all four fungal TPs detected* in vitro* (Figs. S18, S34). The key metabolites with the highest probability scores were: KET-2, KET-3, KET-1, and 2-(3-benzoylphenyl)acrylic acid.

### *Cunninghamella*-processed NSAIDs toxicity evaluation

#### Ecotoxicological analyses

The results showed that biotransformation by *Cunninghamella* reduced the toxicity of both IBU and DCF (Table 4). The most pronounced effect was observed for IBU, where biotransformation led to more than a seven-fold increase in the EC_50_ value compared with the native sample. In the case of DCF, after 15 min of incubation, the EC_50_ value of the biotransformed extract was 1.5 times higher than that of the native extract.

**Table 4:** Toxicity values obtained using the Microtox^®^ assay and the microplate algal bioassay for the tested samples before and after treatment with *Cunninghamella*. Results from the algal bioassay are presented as mean ± SD (*n* = 8).

Tested sample		Microtox^®^ assay		Microplate algal bioassay
		EC_50_ [mg/L]		EC_50_ [µg/mL]
		5 min	15 min	
DCF	+ *Cunninghamella* medium	94.04	72.30	111.88 ± 13.89
	*Cunninghamella* processed	114.40	115.60	nd (> 212.00)
IBU	+ *Cunninghamella* medium	29.28	28.62	134.53 ± 1.31
	*Cunninghamella* processed	161.30	208.20	nd (> 234.00)

The ecotoxicity of the samples was also evaluated by assessing the effect of the extracts on the growth of *P. subcapitata* (Table 4). The results showed that *Cunninghamella* biotransformation contributed to a reduction in the toxicity of both IBU and DCF. The EC_50_ value for the IBU sample before biotransformation was 134.53 µg/mL (the cell count at the highest tested concentration was 33.82% of the control). After treatment with *Cunninghamella* the cell count at the highest tested concentration was 92.63% of control. The EC_50_ value for the DCF sample before biotransformation was 111.88 µg/mL with a cell count of 15.92% of the control at a concentration of 176 µg/mL. After biotransformation, the DCF sample showed a cell count of 84.13% of control at a concentration of 212 µg/mL.

In conclusion, the results of the two ecotoxicity assays indicate that biotransformation by *Cunninghamella* effectively reduces the ecotoxicity of both IBU and DCF. This reduction is particularly pronounced in the algal assay, where the post-biotransformation toxicity is so low that the EC_50_ value could not be determined. However, it should be taken into consideration that extracts used in the toxicity assays contained residual medium-derived components. This could potentially reduce the relative concentration of biotransformation products and consequently lead to an underestimation of their cytotoxic effects, which represents a limitation of the present study. Nevertheless, all samples and control groups in our toxicity studies were processed and analyzed under identical experimental conditions, thereby allowing for a reliable comparative assessment between treatments.

Previous studies have confirmed the ecotoxicity of both IBU and DCF. In the Microtox assay, an EC_50_ of 10.8 mg/L was reported for pure IBU^43^, while other sources indicate values ranging from 11.3 to 19.7 mg/L^44,45^. For DCF, literature data report an EC_50_ of 8.72 mg/L in the Microtox assay^46^. In algal assays, EC_50_ values of 20.50 µg/mL for IBU^47^ and 30.01 µg/mL for DCF^48^ have been reported. It should be noted, however, that in the present study, an extract rather than the pure compound was tested, which may account for differences in observed toxicity.

Only a limited number of studies have investigated the toxicity of DCF TPs. Several of these studies have shown that the photodegradation products of DCF exhibit greater toxicity than the parent compound^49,50^. A comparative study evaluating the toxicity of DCF and DCF-1 in *Mytilus* marine mussels demonstrated that DCF-1 induced greater toxicity and/or more pronounced adverse biological effects than DCF^51^. In our study, a broader range of metabolites was identified beyond DCF-1, which may explain the divergent findings, particularly the observed reduction in ecotoxicity.

In 2020, the results of studies on the ecotoxicity of several pharmaceuticals - including IBU and DCF - as well as their metabolites DCF-1, IBU-2, and carboxyibuprofen (not identified in our study), were published^52^. It was shown that both IBU and DCF were harmful to all tested aquatic organisms. In the *A. fischeri* assay, the ecotoxicity of IBU metabolites was lower than that of the parent compound. For DCF, the ecotoxicity of DCF-1 in this test was comparable to that of the parent compound. In the remaining assays, IBU metabolites also exhibited lower toxicity compared to the parent compound. The toxicity of the DCF metabolite was test-dependent: in the *P. subcapitata* assay, it was more toxic than the parent compound, whereas in the *L. minor* and *D. magna* tests, it was less toxic.

#### Cytotoxicity

Studies on the safety profile of NSAIDs and their metabolites in humans have demonstrated toxicity mainly towards gastric, hepatic, and renal tissues, although neurotoxic and cardiotoxic effects have also been reported^53^.

**Fig. 1:**
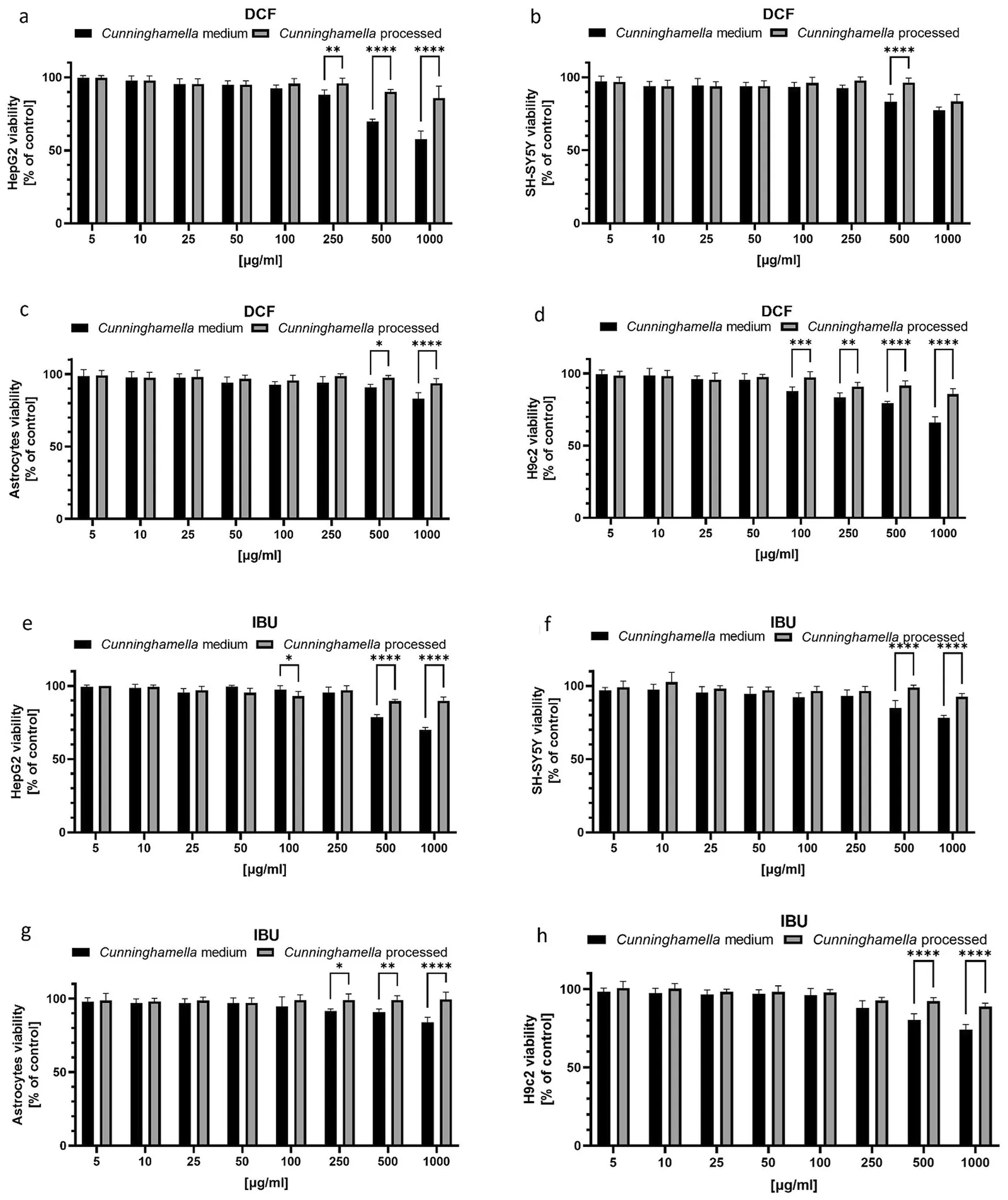
The effect of tested DCF and IBU samples before and after treatment with *Cunninghamella* on the viability of HepG2 (**a**, **e**), SH-SY5Y (**b**, **f**), astrocytes (**c**, **g**), and H9c2 (**d**, **h**) cells. Graphs represent the number of viable cells expressed as percent of control ± SD (*p < 0.05; ** *p* < 0.01; ***p < 0.001; **** *p* < 0.0001).

Both untreated DCF and IBU (Fig. 1) exhibited lower cell viability compared to post-*Cunninghamella* extracts in all investigated cell lines, with significant differences observed at the highest tested concentrations. The most pronounced differences were observed in HepG2 cells, where unprocessed DCF reduced cell viability by approximately 30% and 45%, and unprocessed IBU by approximately 20% and 30% at concentrations of 500 and 1000 µg/mL, respectively. The largest discrepancies in cell viability were also noted for DCF extracts at 1000 µg/mL (astrocytes, H9c2 cells) and 500 µg/mL (SH-SY5Y, H9c2 cells), as well as for IBU extracts at 1000 µg/mL (astrocytes, SH-SY5Y, and H9c2 cells) and 500 µg/mL (SH-SY5Y, H9c2 cells).

As for DCF, Gómez-Lechón et al.^54^ reported cytotoxic effects on HepG2 cells at a concentration of 800 µM, whereas rat hepatocytes exhibited toxicity at a much lower concentration, suggesting the involvement of DCF metabolites in the mechanism of cytotoxicity. Further studies confirmed that DCF metabolites such as 4’-hydroxydiclofenac, diclofenac acyl-β-D-glucuronide, and diclofenac glutathione thioester mediate the induction of acute hepatotoxicity^55,56^. Our results, however, suggest the opposite relationship, with the mixture of DCF derivatives showing lower toxicity compared to the parent compound. This may be explained by the absence of DCF acyl-glucuronide among the detected derivatives. Notably, microorganisms are more likely to catalyze glucosylation reactions rather than glucuronidation, as observed in mammals.

The effect of DCF on SH-SY5Y cells in the LDH assay was evaluated by Cecere et al.^57^, who reported a significant reduction in cell viability after 24-hour incubation at a concentration of 150 µM, while our results indicate a similar reduction at a concentration of 500 µg/mL of the unprocessed DCF extract. Ghosh et al.^53^ demonstrated a significant decrease in proteasome chymotrypsin-like activity as a potential mechanism of DCF toxicity in H9c2 cells. Prolonged exposure to DCF at concentrations of 5 and 10 µM caused a reduction in cell viability of approximately 35% and 55%, respectively. The results obtained by Pashapour et al.^58^ showed a significant reduction in the viability of HepG2 cells after 24-hour incubation with IBU at concentrations of 1 mg/mL and 10 mg/mL compared to the control group, while lower concentrations did not exhibit this effect. The aforementioned data partially support our findings, which, however, indicate even higher cytotoxicity of IBU towards HepG2 cells.

Regarding cardiotoxicity, Gyöngyösi et al.^59^ observed a 13% decrease in the viability of H9c2 cells after 24-hour incubation with IBU at a concentration of 500 µg/mL compared to the control group. This again supports the similarity with our results, although the higher cytotoxicity observed in our assay may be attributed to the use of an extract rather than the pure compound.

#### Mutagenicity

Within the tested concentration range, none of the extracts exhibited mutagenic effects, as the FIB values did not exceed 2, and no significant increase in this parameter with increasing concentration was observed (Table S2). These findings for IBU are consistent with the previous reports^60^. Moreover, our *in vitro* data aligned with *in silico* predictions, as both IBU and its TPs were predicted to be non-toxic with regard to mutagenicity and *in vitro* chromosomal damage (Table S4).

Regarding DCF, our findings are in agreement with previous studies. Domaradzka et al.^61^ reported a lack of mutagenic effect of DCF in the Ames microplate format test using TA98 and TA100 strains, both in the presence and absence of a metabolic activation system. Hartmann et al.^62^ noted that in the Ames tests, diclofenac sodium showed no mutagenic potential at any tested concentration, regardless of the presence of a metabolic activation system. In addition, studies using isolated metabolites − 3′-hydroxy, 4′-hydroxy, 4′,5-dihydroxy, and 5-hydroxy diclofenac -also yielded negative results in the Ames assays.

Contrary to our own and others’ *in vitro* results, the Derek software generated a plausible alert for *in vitro* mutagenicity in bacteria for DCF-7 and DCF-12. Moreover, the presence of diphenylamine and either a 4-alkoxyphenol or 4-aminophenol moiety in some DCF TPs was predicted to result in plausible *in vitro* chromosomal damage (Table S3). To better understand these differing toxicity outcomes, it is important to consider that in silico toxicity predictions were performed for individual DCF metabolites, whereas mutagenicity was evaluated for complex chemical mixtures. Drzymała and Kalka^63^ highlighted that interactions between compounds in the environment can lead to synergistic or antagonistic effects, either amplifying or reducing their individual toxicity levels. Secondly, under ICH M7 guidelines, impurities predicted to be mutagenic using in silico methodologies must be further assessed using the bacterial reverse mutation assay, followed by expert review^64^.

#### Endocrine disrupting properties

Prior to biotransformation, IBU and DCF did not exhibit agonistic estrogenic activity (Table 5). *Cunninghamella* biotransformation led to the emergence of estrogenic properties. In their native form, both compounds showed antagonistic estrogenic activity, as well as antagonistic androgenic activity. Following biotransformation, IBU and DCF lost both antagonistic estrogenic and androgenic activities. No agonistic androgenic activity was detected for either compound. This is consistent with literature data showing that IBU and DCF do not exhibit agonistic activity toward estrogen or androgen receptors, but instead display anti-estrogenic and anti-androgenic effects^65^.

**Table 5:** Estrogenic and androgenic activities of samples before and after treatment with *Cunninghamella* (E2-17β-estradiol; HT-4-hydroxytamoxifen; DHT-5α-dihydrotestosterone; FLU-flutamide; IR “+”-the sample exhibits the respective activity; IR “−”- the sample does not exhibit the respective activity; nd (not determined) – the concentration of estrogenic or androgenic substances was below 10^− 10^ M for estrogens (YES) or below 10^− 9^ M for androgens (YAS) compared to the standard).

Tested sample		Estrogenic activity				Androgenic activity			
		Agonistic estrogenic activity		Antagonistic estrogenic activity		Agonistic androgenic activity		Antagonistic androgenic activity	
		I_R_	EEQ (ng E2/mg extract)	I_R_	aEEQ (ng HT/mg extract)	I_R_	AEQ (ng DHT/mg extract)	I_R_	aAEQ (ng FLU/mg extract)
DCF	+ *Cunninghamella* medium	–	nd	+	118.02	–	nd	+	0.12
	*Cunninghamella* processed	+	3.33 × 10^− 4^	–	nd	–	nd	–	nd
IBU	+ *Cunninghamella* medium	–	nd	+	268.41	–	nd	+	0.84
	*Cunninghamella* processed	+	1.69 × 10^− 3^	–	nd	–	nd	–	nd

Regarding the endocrine activity of metabolites of IBU and DCF, for IBU there are no published studies specifically addressing the receptor-mediated endocrine activity of IBU’s main metabolites. However, 4′-hydroxydiclofenac has been investigated and shown to exhibit endocrine-disrupting properties^66^. The results obtained from the YES assay were consistent with our findings – only the DCF metabolite exhibited agonistic activity towards the estrogen receptor. Studies have also demonstrated that DCF is a stronger antagonist of the estrogen receptor than its metabolite. This aligns with our results, where the extract obtained after biotransformation with *Cunninghamella* lost its antagonistic activity toward the estrogen receptor.

With regard to androgen receptor activity, in the YAS assay researchers reported strong agonistic activity of 4′-hydroxydiclofenac. In contrast, in our experiments, both the crude extract and the extract after biotransformation lacked such activity. As for antagonistic activity toward the androgen receptor, both compounds exhibited such effects, with stronger activity observed for DCF-1. However, our results differ in that the extract after biotransformation lost its antagonistic activity towards AR. It is important to note that the cited study tested only a single DCF metabolite, whereas our experiments involved a mixture of eleven compounds. This may explain the observed differences in (anti)androgenic activity. The researchers also showed that both DCF and its metabolite DCF-1 exhibited significant (anti)-glucocorticoid and anti-thyroid hormonal activities^66^.

Notable differences were observed when comparing our* in vitro* results with *in silico *predictions. While the *in vitro *experiments indicated (anti)endocrine activity for certain extracts and metabolites, the *in silico* model did not predict any endocrine-disrupting effects for either the parent compounds or their metabolites. This discrepancy underscores the importance of experimental validation, as computational models may not fully capture the complexity of receptor-mediated interactions.

#### *In silico* toxicity

The toxicity evaluation of the parent compounds as well as their TPs is summarized in Tables S3-S5 (Supplementary Material). Derek Nexus indicated that the tested NSAIDs and all their TPS were considered safe with respect to neurotoxicity, estrogenicity, and teratogenicity. IBU and its TPs were also predicted to be non-toxic with regard to mutagenicity, carcinogenicity, and *in vitro* chromosomal damage. However, the parent compound and its biodegradation products IBU-1, IBU-2, IBU-3, and IBU-4 exhibited structural hepatotoxicity alerts. Moreover, Derek predicted nephrotoxicity for derivatives IBU-2, IBU-4, and IBU-5b.

DCF-7 and DCF-12 triggered a plausible alert for *in vitro* mutagenicity. Moreover, the presence of diphenylamine and either a 4-alkoxyphenol or 4-aminophenol moiety in some DCF TPs was predicted to result in *in vitro* chromosomal damage. Additionally, DCF and the majority of its TPs were anticipated to induce hepatotoxicity and nephrotoxicity.

Considering the third compound, KET and its TPs did not produce any alerts related to *in vitro* mutagenicity or chromosomal damage. However, KET and all its TPs, except for KET-4, triggered a plausible alert for carcinogenicity. Moreover, the presence of 2-arylacetic or 3-arylpropionic acid moieties, as well as aryl or fulvenyl acetic or 2-propionic acid derivative moieties, indicated specific alerts, representing potential toxophores. These structural features reinforce the positive hepatotoxicity and nephrotoxicity potential of the parent compound and all its metabolites (except for KET-1f).

## Conclusion

This study demonstrates that *Cunninghamella* species can effectively biotransform diclofenac and ibuprofen, leading to a significant reduction in their ecotoxicity. Cytotoxicity assessment using human and rat cell lines revealed that untreated NSAID extracts exhibited higher toxicity compared to post-biotransformation extracts, indicating that fungal biotransformation decreases toxicity toward liver, neuronal, astrocytic, and cardiomyocyte cells. Moreover, biotransformation led to the loss of antagonistic estrogenic and androgenic activities originally present in the parent compounds, while the fungal transformation products exhibited only weak estrogenic agonism. In contrast, ketoprofen showed negligible susceptibility to fungal degradation and was therefore not further analyzed for toxicity.

In conclusion, fungal biotransformation by *Cunninghamella* appears to be a promising strategy for reducing the environmental toxicity of certain NSAIDs. Our results extend the current scope of NSAIDs biotransformation products’ safety assessment, covering not only ecotoxicity but also their cytotoxicity, mutagenic potential, and endocrine-disrupting properties. The generation of transformation products with altered biological activity underscores the importance of combining analytical identification with comprehensive toxicological assessment, which has been limited in previous studies. The results presented in this paper contribute to filling this gap by demonstrating the importance of parallel chemical and toxicological characterization of NSAID transformation products. Since ketoprofen was not biotransformed by tested *Cunninghamella* strains, further studies should include other strains or consortia of microorganisms, as well as optimization of culture conditions to increase the applicability of microbiological methods for NSAIDs removal. In view of the high biotransformation efficiencies achieved for DCF and IBU, together with the favorable changes in the toxicological profile of the resulting derivatives, all investigated *Cunninghamella* strains appear to be promising candidates for further studies on the removal of other pharmaceuticals and their potential application in aquatic environmental remediation systems.

## Materials and methods

### Reagents and chemicals

Diclofenac (CAS No. 15307-86-5), ibuprofen (CAS No. 15687-27-1), and ketoprofen (CAS No. 22071-15-4) were purchased from Merck (Darmstadt, Germany). Unless otherwise stated, all other reagents and chemicals used in this study were also obtained from Merck.

### Microbial biotransformation

#### *Cunninghamella spp.*

The biotransformation procedures followed previously described protocols, with minor modifications^12^. Strains of *Cunninghamella echinulata* (DSM 1905), *Cunninghamella blakesleeana* (DSM 1906), and *Cunninghamella elegans* (DSM 1908) were obtained from the Deutsche Sammlung von Mikroorganismen und Zellkulturen (DSMZ, Braunschweig, Germany) and cultured on 3% potato dextrose agar (PDA; Merck, Darmstadt, Germany) at 27 °C for 7 days. Subsequently, a liquid biotransformation medium (25 mL) comprising 5 g/L peptone from soybean, yeast extract, NaCl, K₂HPO₄, and 20 g/L glucose (Chempur, Poland) was inoculated with 1 mL of a spore suspension harvested from PDA plates using sterile water. After 48 h of incubation, the cultures were treated with 12.5 mg of the target compound dissolved in DMSO and shaken for a further 7 days. Controls lacking the substrate or fungal inoculum were processed in the same way. Each condition was performed in triplicate in two independent experiments. The aqueous phase was separated from the fungal biomass by filtration of the biotransformation mixtures. The filtrates were extracted with dichloromethane (Chempur, Piekary Śląskie, Poland), dried over anhydrous sodium sulfate, evaporated to dryness under reduced pressure, and redissolved in methanol. The obtained samples were subsequently analyzed using ultra-high-performance liquid chromatography coupled with mass spectrometry (UHPLC/MS). The UHPLC-QTof system consisted of a Waters Acquity I-Class Plus coupled to a Waters Synapt XS mass spectrometer (electrospray ionization mode ESI-QTof). Chromatographic separations were carried out using the Acquity UPLC BEH (bridged ethyl hybrid) C_18_ column; 2.1 × 100 mm, and 1.7 μm particle size, equipped with Acquity UPLC BEH C_18_ VanGuard pre-column; 2.1 × 5 mm, and 1.7 μm particle size. The column was maintained at 40 °C and eluted under following conditions: isocratic elution with 95% of eluent A over 0.5 min, afterwards linear gradient elution from 95% to 0% of eluent A over 9.5 min, 100% of eluent B over 3.5 min, at a flow rate of 0.3 mL min^− 1^. Next, the column was reconditioned: linear gradient from 0% to 95% of A over 0.5 min and flushing the column with 95% of A over 1.0 min, at a flow rate of 0.3 mL min^− 1^. Eluent A: water/formic acid (0.1%, v/v); eluent B: acetonitrile/formic acid (0.1%, v/v). Chromatograms were recorded using Waters eλ PDA detector. Spectra were analyzed in 200–800 nm range with 1.2 nm resolution and sampling rate 20 points s^− 1^. MS detection settings of Waters Synapt XS mass spectrometer were as follows: source temperature 150 °C, desolvation temperature 250 °C, desolvation gas flow rate 600 L h^− 1^, cone gas flow 100 L h^− 1^, capillary potential 3.00 kV, cone potential 30 V. Nitrogen was used for both nebulizing and drying gas. The data were obtained in a resolution MS^E^ (full MS scan with simultaneous data independent fragmentation) and daughters scan modes ranging from 50 to 1000 *m/z* in time 0.2 s intervals. Leu-enkephalin was used as mass reference. Analyses were carried out in positive and negative ionization modes. Collision activated dissociations (CAD) analyses were carried out with the energy ramp from 10 eV to 50 eV. Argon was used as collision gas. Data acquisition and analysis software were MassLynx v4.2 and MS^E^ Data Viewer v2.0 (Waters). Simultaneously, dried extracts were dissolved in 1 mL of DMSO for use in toxicity assays.

#### *Aspergillus niger*

The ability of the filamentous fungus *A. niger* to remove tested NSAIDs was evaluated according to modified protocol described by Hussain et al.^67^. *A. niger* (DSM 821, DSMZ, Braunschweig, Germany) was cultured and subjected to biotransformation under conditions analogous to those described for *Cunninghamella* strains, with the following modifications: incubation was conducted at 30 °C and the biotransformation medium consisted of 10 g/L glucose, 5 g/L casein peptone, 5 g/L KH₂PO₄, 5 g/L yeast extract, 5 g/L NaCl (Chempur, Poland), and 5 mL/L glycerol (Pharma Cosmetic, Kraków, Poland). All experiments, including appropriate controls, were performed in triplicate in two independent experiments. Filtration, extraction, and chromatographic analyses were performed in accordance with Section *Microbial biotransformation: Cunninghamella spp*.

#### *Trametes versicolor*

*Trametes versicolor* (DSM 11309, DSMZ, Braunschweig, Germany), was cultured for 7 days at 25 °C in the dark on malt extract peptone agar (DSMZ medium 90, pH 5.6). Degradation experiments were conducted following the methods of previous studies^12,68,69^. A nitrogen-rich medium (NRM) supplemented with a mineral salt solution was inoculated with agar plugs containing mycelium and incubated for 5 days. The mycelial mass was homogenized and diluted in NaCl to prepare a suspension. Pellets were generated by inoculating this suspension into NRM and incubating for 7 days. Degradation experiments were conducted with 10 g wet mycelial pellets per 100 mL of NRM. Test compounds (12.5 mg in DMSO) were added to biotransformation medium and incubated. Filtration, extraction, and chromatographic analyses were performed as described in Section *Microbial biotransformation: Cunninghamella spp.* Experiments were carried out in triplicate and repeated twice. Control setups consisted of cultures without test compounds and flasks containing only the medium with the tested substances.

### Cytochrome P450 inhibition study

1-Aminobenzotriazole (ABT), a known inhibitor of CYP450 enzymes, was used to differentiate between CYP-mediated and non-CYP-mediated biotransformation pathways. Fungal strains were cultured in the standard biotransformation medium. After a 48-hour incubation, *Cunninghamella* cultures were treated with (ABT) at a final concentration of 3 mM (prepared from a 96% ethanol stock solution) for 1 h at 27 °C. Subsequently, the tested NSAIDs (12.5 mg per 25 mL medium) in DMSO, were added. Cultures were then incubated for 7 days. Following incubation, biotransformation, filtration, extraction, and chromatographic analyses were performed as previously described (Section *Microbial biotransformation: Cunninghamella spp.*). Control samples included substrate-only, inhibitor-only, and combined inhibitor-substrate groups. Due to the negligible removal of KET by *Cunninghamella* species, this compound was not included in this study.

### *In vitro* toxicity evaluation of post-biotransformation samples

Two types of extracts were tested in toxicity assays: those from NSAIDs biotransformed by *Cunninghamella* species, and those from untransformed NSAIDs incubated in the biotransformation medium. Due to differences in the biodegradation efficiency among *Cunninghamella* strains, the extract obtained using the most effective strain for each tested NSAID was selected for further toxicological analyses (*C. echinulata* for DCF and *C. elegans* for IBU). Due to the negligible removal of KET by *Cunninghamella* species, this compound was excluded from further toxicity testing.

### Ecotoxicological analyses

#### Microtox^®^ assay

The toxicity of the samples was evaluated using the bioluminescence inhibition test with *Aliivibrio fischeri* (Microtox test). Measurements were taken using the Microtox Model 500 Analyzer (Modern Water, New Castle, USA). The assay followed the Microtox Basic Test protocol provided by the manufacturer. Freeze-dried *A. fischeri* cultures were reconstituted with a 0.01% sodium chloride solution. Reagents, including osmotic adjustment solution (OAS, 22% NaCl) and diluent were supplied by Modern Water. Serial dilutions of test compounds in DMSO were prepared using a twofold dilution factor. The incubation mixtures contained 25 µL of test solution, 2475 µL of 2% sodium chloride diluent, and 125 µL of OAS. Final test concentrations ranged from 29.46 to 235.70 mg/L for all extracts. The DMSO concentration was 0.95% v/v. Preliminary trials established optimal concentration ranges for EC_50_ determination, defined as the concentration causing a 50% reduction in bioluminescence. Toxicity was assessed after 5 and 15 min of incubation at 15 °C (± 0.5 °C). Data analysis was performed using MicrotoxOmni™ software (Modern Water, New Castle, USA).

#### Algal growth inhibition assay

The *Pseudokirchneriella subcapitata* growth inhibition test was performed according to OECD guideline^70^, with adaptations for 96-well microplates based on previously described protocol^71^. Algae *P. subcapitata* (ATCC 22662) were obtained from the American Type Culture Collection (ATCC), Manassas, VA, USA. *P. subcapitata* was cultured in OECD medium supplemented with 2% (w/v) agar and stored at 4 °C. Pre-cultures and cultures were maintained in OECD medium at 25 °C under continuous cool-white fluorescent light (5 000 lx), and shaken continuously at 100 rpm in a culture chamber. Algal cultures in the exponential growth phase were used to inoculate the test samples and controls. Algal cell density was determined with an automated cell counter (Bio-Rad). The toxicity test was carried out in 96-well cell culture plates. Seven test concentrations were prepared, each in four replicates, and two independent experiments were performed. An appropriate geometric series was used, in which each successive concentration was approximately half of the previous one. The final concentrations of the tested samples in each well of the culture system were as follows: 352, 176, 88, 44, 22, 11, and 5.5 µg/mL for DCF + *Cunninghamella* medium; 212, 106, 53, 26.5, 13.25, 6.63, and 3.31 µg/mL for *Cunninghamella*-processed DCF samples; 129, 64.5, 32.25, 16.13, 8.06, 4.03, and 2.02 µg/mL for IBU + *Cunninghamella* medium; and 234, 117, 58.5, 29.25, 14.63, 7.31, and 3.66 µg/mL for *Cunninghamella*-processed IBU samples. The above-mentioned final concentrations were based on the initial concentrations of the extracts obtained after evaporation, as well as on the safe concentration of the solvent (DMSO). Peripheral wells were filled with sterile water, negative control wells contained OECD medium and the solvent. DMSO was used at the final concentration determined in preliminary experiments, which was shown not to affect the growth of *P. subcapitata.* Potassium dichromate (K_2_Cr_2_O_7_) was used as a reference compound and tested every two to three months. The algal suspensions were incubated at 25 °C under continuous cool-white luminescent light for 72 h. At the end of the exposure, cell density was measured using a plate reader (SpectraMax iD3, Molecular Devices, USA) at 430 nm, based on a standard calibration curve. Median effective concentration values (EC_50_) defined as the concentration causing 50% inhibition of the growth rate after 72 h, were estimated for each tested toxicant using Prism 9.0 software (Graphpad Inc., CA, USA).

#### Cytotoxicity assessment

Human hepatocellular carcinoma HepG2 (ATCC HB-8065; ATCC, Manassas, VA, USA), human neuroblastoma SH-SY5Y (CRL-2266; ATCC), immortalized human astrocytes (P10251-IM; Innoprot, Spain), and rat cardiomyocytes H9c2 (CRL-1446; ATCC) cell lines were cultured in media recommended by the manufacturers. Cells were seeded in 96-well plates at a density of 1 × 10⁴ cells/well for HepG2 and H9c2, 2 × 10⁴ cells/well for SH-SY5Y, and 1 × 10^5^ cells/well for astrocytes, followed by a 24-hour incubation period. The extracts were administered at final concentrations ranging from 5 to 1000 µg/mL, with 0.1% DMSO used as the vehicle control, and incubated for an additional 24 h. Following treatment, the MTT reagent (3-(4,5-dimethylthiazol-2-yl)-2,5-diphenyltetrazolium bromide) was added, and the cells were incubated for 4 h to facilitate formazan production. The formazan crystals were dissolved in DMSO, and absorbance was measured at 570 nm. Experiments were performed in triplicate, with each condition run in duplicate. Statistical analysis was conducted using two-way ANOVA with Šídák’s multiple comparisons test, performed in GraphPad Prism 9.0.

#### Mutagenicity evaluation

The mutagenic potential of the extracts was assessed using the Ames MPF assay with the following bacterial strains: *Salmonella typhimurium* TA98, TA100, TA1535, and TA1537; and *Escherichia coli* WP2 uvrA[pKM101] (Xenometrix, Allschwil, Switzerland). The assay was performed according to the manufacturer’s instructions. First, bacterial strains were inoculated into a growth medium and incubated overnight at 37 °C with shaking. The next day, the cultures were diluted in exposure medium and treated with the extracts in 24-well plates for 90 min at 37 °C with agitation. The samples were tested at three final concentrations, selected based on preliminary cytotoxicity experiments using the TA98 strain. Following preincubation, the cultures were diluted in indicator medium and transferred to 48 wells on 384-well plates, followed by a 48-h incubation. The presence of revertant wells was determined by a color change (yellow), and results were compared to negative controls containing solvent only. Positive controls were strain-specific mutagens, including: 2-nitrofluorene (2-NF) for TA98 (2 µg/mL), 4-nitroquinoline-N-oxide (4-NQO) for TA100 (0.1 µg/mL), N4-aminocytidine (N4-ACT) for TA1535 (100 µg/mL), 9-aminoacridine (9-AAc) for TA1537 (15 µg/mL), 4-NQO for *E. coli* (2 µg/mL). Pure DMSO was used as the negative control. The mutagenic potential of the test samples was determined according to the manufacturer’s instructions, which involve assessing the fold increase above the zero-dose baseline (defined as the mean of the negative control plus one standard deviation) and evaluating the presence of a dose-dependent response. The results were interpreted as follows: a sample was classified as positive if it showed more than a 2-fold induction over the baseline (fold induction over baseline, FIB) at more than one concentration, along with a clear dose-response relationship. In contrast, samples that did not show a > 2-fold increase or lacked evidence of a dose-response were considered negative.

#### Endocrine-disrupting potential assessment

The estrogenic, antiestrogenic, androgenic, and antiandrogenic properties of the extracts were assessed using the XenoScreen XL YES/YAS assay (Xenometrix AG, Allschwil, Switzerland). This assay employs *Saccharomyces cerevisiae* strains stably expressing human estrogen (hERα) and androgen (hAR) receptors, along with genetic constructs encoding the *lacZ* reporter gene for β-galactosidase. Upon ligand binding, the appropriate receptor activates transcription of the reporter gene via specific response elements, resulting in β-galactosidase-mediated conversion of the yellow substrate chlorophenol red-β-D-galactopyranoside into a red product. Expression of β-galactosidase is directly proportional to the activating (agonistic) or inhibiting (antagonistic) activity of the tested compounds. Color change and yeast growth were quantitatively measured using a microplate reader (SpectraMax iD3, Molecular Devices, USA) at 570 and 690 nm, respectively. Agonistic and antagonistic activities for both estrogen and androgen receptors were evaluated. In addition, the assay uses a reduction in light absorbance at 690 nm as an indicator of compound-induced toxicity in yeast cells. The procedure was performed according to the manufacturer’s protocol, using standardized reagents and materials supplied in the kit. Eight serial half-logarithmic dilutions were prepared from individual extract stock solutions (50 mg/mL in DMSO), resulting in final well concentrations of 500, 158.2, 50.1, 15.9, 5.0, 1.6, 0.5, and 0.2 µg/mL. Standard controls included 17-β estradiol for YES agonists, 4-hydroxytamoxifen for YES antagonists, 5α-dihydrotestosterone for YAS agonists, and flutamide for YAS antagonists. The manufacturer’s criteria were applied to evaluate cell viability and receptor-specific activity. Data analysis included the calculation of the induction ratio (IR) based on spectrophotometric readings, as well as the quantitative derivation of estrogen equivalent (EEQ), antiestrogen equivalent (aEEQ), androgen equivalent (AEQ), and antiandrogen equivalent (aAEQ) values. These parameters represent the concentration of either estradiol or dihydrotestosterone required to elicit a response equivalent to that of the tested sample.

#### *In silico* toxicity

The in silico toxicity potential of NSAIDs TPs was evaluated using Derek Nexus (v. 6.1.1, Lhasa Limited, Leeds, UK), an expert rule-based software system for toxicity prediction. This commercial tool applies a comprehensive set of alerts and mechanistic reasoning rules-developed and curated by expert toxicologists-to assess the toxicological hazards of chemical structures across a range of endpoints. Derek Nexus provides qualitative, non-numeric likelihood predictions regarding the potential toxicity of compounds, using a scale of descriptors: certain, probable, plausible, equivocal, doubted, improbable, impossible, contradicted, and open. TPs were evaluated for potential hepatotoxicity and nephrotoxicity in mammals, neurotoxicity, estrogenicity, mutagenicity *in vitro* in bacteria, chromosome damage *in vitro* in mammals, carcinogenicity, and teratogenicity using the Derek Nexus system. Each endpoint prediction was generated by comparing the structure of each metabolite to a curated database of structure-toxicity associations and mechanistic reasoning frameworks embedded within the software.

#### *In silico* metabolism prediction

The prediction of metabolites derived from the tested NSAIDs was carried out using MetaSite software, version. 6.0.1 (Molecular Discovery Ltd., UK). MetaSite is a computational platform designed to predict the most likely metabolic transformations of candidate compounds, primarily focusing on phase I hepatic metabolic, such as reactions mediated by CYP450 enzymes and flavin-containing monooxygenases (FMOs). The software employs a physics-informed algorithm that integrates both thermodynamic factors (enzyme-substrate recognition) and kinetic parameters (reaction transformation rates) to simulate interactions between substrates and metabolic enzymes. For each compound, MetaSite generates the structures of predicted metabolites based on identified sites of metabolism, and assigns a probability score to each, with the highest likelihood represented as 100%.

## Supplementary Information

Below is the link to the electronic supplementary material.

Supplementary Material 1

## Data Availability

All data generated or analyzed during this study are included in this published article and its supplementary information files. Additional raw data are available from the corresponding author upon reasonable request.
