# Supplementary material for: Biotransformation of common NSAIDs by Cunninghamella species and resulting metabolite toxicity
Source: Sci Rep. 2026 Jun 18;16:27837. doi: 10.1038/s41598-026-58519-6 (PMC13547222; doi:10.1038/s41598-026-58519-6)
Supplement: Supplementary file 1 [file 41598_2026_58519_MOESM1_ESM.docx]

**Biotransformation of common NSAIDs by *Cunninghamella* species and resulting metabolite toxicity**

Fig. S1. HPLC chromatogram of the extract of the incubation mixture of DCF with *C. echinulata*.

Fig. S2. HPLC chromatogram of the extract of the incubation mixture of DCF with *T. versicolor*.

Fig. S3. HPLC chromatogram of the extract of the incubation mixture of DCF with *C. elegans*.

Fig. S4. HPLC chromatogram of the extract of the incubation mixture of DCF with *A. niger*.

Fig. S5. MS/MS spectrum of metabolite DCF-1.

Fig. S6. MS/MS spectrum of metabolite DCF-2.

Fig. S7. MS/MS spectrum of metabolite DCF-3.

Fig. S8. MS/MS spectrum of metabolite DCF-4.

Fig. S9. MS/MS spectrum of metabolite DCF-5.

Fig. S10. MS/MS spectrum of metabolite DCF-6.

Fig. S11. MS/MS spectrum of metabolite DCF-7.

Fig. S12. MS/MS spectrum of metabolite DCF-8.

Fig. S13. MS/MS spectrum of metabolite DCF-9.

Fig. S14. MS/MS spectrum of metabolite DCF-10.

Fig. S15. MS/MS spectrum of metabolite DCF-11.

Fig. S16. *Cunninghamella* biotransformation. Cytochrome P450 inhibition study by 1-aminobenzotriazole (ABT), experiment with DCF and *C. echinulata*.

Fig. S17. *In silico* biotransformation of DCF. Main metabolite structures and graphs representing scores for each metabolite. Metabolite IDs correspond to those in Table 2. IDs expressed in Roman numerals represent metabolites predicted *in silico* but not observed in experimental studies.

Fig. S18. Venn diagrams comparing metabolites of DCF (a), IBU (b) and KET (c) identified experimentally during fungal biotransformation and predicted *in silico* by MetaSite software. The fungal strains were assigned numerical designations (*C. echinulata* – 1, *C. blakesleeana* – 2, *C. elegans* – 3, *A. niger* – 4, *T. versicolor* – 5).

Fig. S19. HPLC chromatogram of the extracts of the incubation mixture of IBU with *C. elegans*.

Fig. S20. HPLC chromatogram of the extract of the incubation mixture of IBU with *T. versicolor*.

Fig. S21. MS/MS spectrum of metabolite IBU-1.

Fig. S22. MS/MS spectrum of metabolite IBU-2.

Fig. S23. MS/MS spectrum of metabolite IBU-3.

Fig. S24. MS/MS spectrum of metabolite IBU-4.

Fig. S25. MS/MS spectrum of metabolite IBU-5.

Fig. S26. *Cunninghamella* biotransformation. Cytochrome P450 inhibition study by 1-aminobenzotriazole (ABT), experiment with IBU and *C. elegans*.

Fig. S27. *In silico* biotransformation of IBU. Main metabolite structures and graphs representing scores for each metabolite. Metabolite IDs correspond to those in Table 3. IDs expressed in Roman numerals represent metabolites predicted *in silico* but not observed in experimental studies.

Fig. S28. HPLC chromatogram of the extracts of the incubation mixture of KET with *C. elegans*.

Fig. S29. HPLC chromatogram of the extract of the incubation mixture of KET with *T. versicolor*.

Fig. S30. MS/MS spectrum of metabolite KET-1.

Fig. S31. MS/MS spectrum of metabolite KET-2.

Fig. S32. MS/MS spectrum of metabolite KET-3.

Fig. S33. MS/MS spectrum of metabolite KET-4.

Fig. S34. *In silico* biotransformation of KET. Main metabolite structures and graphs representing scores for each metabolite. Metabolite IDs correspond to those in Table S1. IDs expressed in Roman numerals represent metabolites predicted *in silico* but not observed in experimental studies.

Table S1. Hypothesized structures, molecular formulas, and selected parameters of KET biotransformation products obtained in the tested fungal strains (*C.* *echinulata* *–*1, *C. blakesleeana – 2, C. elegans – 3, A. niger – 4, T. versicolor – 5).*

| **Peak ID** | **Chemical structure** | **Formula** | **R_t_ (min)** | **[M+H]^+^/**  **[M+H]^-^** | **Major daughter ions** | **MetaSite prediction** | **Proposed biotransformation reaction** | **Fungal strain** | **References** |
| --- | --- | --- | --- | --- | --- | --- | --- | --- | --- |
| KET |  | C_16_H_14_O_3_ | 6.30 | 253 | 209 | - | - | **-** | ^S 1,S 2^ |
| KET-1 |  | C_16_H_14_O_4_ | 5.17 | 269 | 252 | + | hydroxylation | 3 | - |
| KET-2 |  | C_16_H_14_O_4_ | 6.54 | 269 | 225 | + | aromatic hydroxylation | 5 | ^S 1,S 3^ |
| KET-3 |  | C_16_H_14_O_4_ | 6.86 | 269 | 254, 225, 209, 177, 149, 121 | + | aromatic hydroxylation | 5 | ^S 3^ |
| KET-4 |  | C_16_H_16_O_3_ | 7.52 | 255 | 211 | + | ketone reduction | 4,5 | ^S 1,S 3^ |

Table S2. Mutagenic activity of Cunninghamella-treated and untreated samples tested with the Ames assay (Mean ± SD - mean value ± standard deviation (n = 3), FIB – fold induction over baseline).

| **Extract ID** | | **Concentration [µg/mL]** | **Bacterial strain** | | | | |
| --- | --- | --- | --- | --- | --- | --- | --- |
|  |  |  | ***S. typhimurium*** | | | | ***E.coli*** |
|  |  |  | **TA98** | **TA100** | **TA1535** | **TA1537** |  |
|  |  |  | **Mean ± SD (FIB)** | | | | |
| Negative control | | - | 0.9 ± 0.8 | 5.3 ± 2.4 | 1.5 ± 1.1 | 1.0 ± 1.3 | 1.0 ± 2.0 |
| Positive control | | - | 47.6 ± 0.5 (28.0) | 47.3 ± 1.0 (6.1) | 48.0 ± 0.0 (18.5) | 48.0 ± 0.0 (20.9) | 45.8 ± 5.7 (22.9) |
| DCF | + *Cunninghamella* medium | 31.2 | 0.3 ± 0.6 (0.3) | 5.7 ± 4.9 (0.8) | 1.7 ± 0.6 (0.4) | 0.3 ± 0.6 (0.3) | 0.7 ± 0.6 (0.3) |
|  |  | 62.5 | 0.7 ± 0.6 (0.6) | 3.0 ± 1.0 (0.4) | 1.7 ± 0.6 (0.4) | 1.3 ± 0.6 (1.3) | 1.7 ± 2.1 (0.8) |
|  |  | 125 | 0.3 ± 0.6 (0.3) | 4.0 ± 2.0 (0.6) | 0.3 ± 0.6 (0.1) | 0.3 ± 0.6 (0.3) | 0.3 ± 0.6 (0.2) |
|  | *Cunninghamella* processed | 500 | 1.0 ± 1.0 (1.0) | 6.7 ± 3.8 (0.8) | 0.3 ± 0.6 (0.3) | 4.7 ± 4.5 (1.7) | 0.3 ± 0.6 (0.2) |
|  |  | 1000 | 1.7 ± 1.5 (1.7) | 7.0 ± 1.0 (0.8) | 0.3 ± 0.6 (0.3) | 0.3 ± 0.6 (0.1) | 0.7 ± 1.2 (0.4) |
|  |  | 2000 | 1.0 ± 1.7 (1.0) | 7.3 ± 1.5 (0.9) | 1.7 ± 1.2 (1.4) | 1.0 ± 1.0 (0.3) | 1.0 ± 1.0 (0.1) |
| IBU | + *Cunninghamella* medium | 125 | 1.0 ± 1.0 (0.4) | 5.6 ± 1.5 (1.0) | 2.0 ± 1.0 (0.8) | 1.3 ± 1.5 (1.3) | 1.0 ± 1.0 (0.6) |
|  |  | 250 | 1.0 ± 0.0 (0.4) | 4.0 ± 0.0 (0.7) | 1.0 ± 0.0 (0.4) | 0.3 ± 0.6 (0.3) | 0.3 ± 0.6 (0.2) |
|  |  | 500 | 0.3 ± 0.6 (0.1) | 1.7 ± 1.2 (0.3) | 1.7 ± 2.1 (0.7) | 0.3 ± 0.6 (0.3) | 0.3 ± 0.6 (0.2) |
|  | *Cunninghamella* processed | 500 | 1.7 ± 0.6 (1.7) | 7.0 ± 4.4 (0.7) | 1.3 ± 1.5 (0.7) | 1.0 ± 0.0 (0.8) | 2.3 ± 2.1 (1.1) |
|  |  | 1000 | 1.7 ± 1.5 (1.7) | 5.7 ± 1.5 (0.6) | 1.7 ± 0.6 (0.9) | 1.3 ± 1.5 (1.2) | 2.3 ± 0.6 (1.1) |
|  |  | 2000 | 1.7 ± 1.2 (1.7) | 6.7 ± 3.1 (0.7) | 1.0 ± 1.0 (0.5) | 0.3 ± 0.6 (0.3) | 1.3 ± 2.3 (0.1) |

Table S3. *In silico* toxicity assessment of DCF and its transformation products (TPs) using Derek Nexus (NA - „No alerts”; structural alerts codes are given in brackets: halogenated benzene (035); polyhalogenated aromatic (116); quinone, precursor or analogue (327); 4-alkoxyphenol or 4-aminophenol (470); aryl or fulvenyl acetic or 2-propionic acid derivative (801); p-aminophenol or derivative (619); 2-arylacetic or 3-arylpropionic acid (620); p-alkylphenol or derivative (666); α,β-unsaturated aldehyde, ketone or imine (743); aryl or fulvenyl acetic or 2-propionic acid derivative (801); diphenylamine (1006)).

| **DCF and its TPs** | **Chemical structure** | **Hepatotoxicity**  **in mammal** | **Nephrotoxicity**  **in mammal** | **Neurotoxicity** | **Estrogenicity** | **Mutagenicity**  ***in vitro***  **in bacterium** | **Chromosome damage *in vitro* in mammal** | **Carcinogenicity** | **Teratogenicity** |
| --- | --- | --- | --- | --- | --- | --- | --- | --- | --- |
| **DCF** |  | Equivocal  (620) | Probable  (035; 801) | NA | NA | Inactive | NA | Plausible  (1006) | NA |
| **DCF-1** |  | Plausible  (620) | Plausible  (035; 801) | NA | NA | Inactive | Plausible  (1006) | NA | NA |
| **DCF-2** |  | Plausible  (620) | Plausible  (035; 801) | NA | NA | Inactive | Plausible  (1006) | NA | NA |
| **DCF-3** |  | Plausible  (619; 620) | Plausible  (035; 801) | NA | NA | Inactive | Plausible  (470; 1006) | NA | NA |
| **DCF-4** |  | Plausible  (620) | Plausible  (035; 801) | NA | NA | Inactive | Plausible  (1006) | NA | NA |
| **DCF-5** |  | NA | Equivocal  (035) | NA | NA | Inactive | NA | NA | NA |
| **DCF-6** |  | Plausible  (620) | Plausible  (035; 801) | NA | NA | Inactive | Plausible  (1006) | NA | NA |
| **DCF-7** |  | NA | NA | NA | NA | Plausible  (327) | NA | Plausible  (743) | NA |
| **DCF-8** |  | Plausible  (620) | Plausible  (801) | NA | NA | Inactive | Plausible  (1006) | NA | NA |
| **DCF-9a** |  | NA | Plausible  (035; 801) | NA | NA | Inactive | Plausible  (1006) | NA | NA |
| **DCF-9b** |  | Plausible  (619) | Plausible  (035; 801) | NA | NA | Inactive | Plausible  (470; 1006) | NA | NA |
| **DCF-9c** |  | Plausible  (666) | Plausible  (035; 801) | NA | NA | Inactive | Plausible  (1006) | NA | NA |
| **DCF-9d** |  | NA | Plausible  (035; 801) | NA | NA | Inactive | Plausible  (1006) | NA | NA |
| **DCF-9e** |  | NA | Plausible  (035; 801) | NA | NA | Inactive | Plausible  (1006) | NA | NA |
| **DCF-9f** |  | NA | Plausible  (035; 801) | NA | NA | Inactive | Plausible  (1006) | NA | NA |
| **DCF-9g** |  | NA | Plausible  (035; 801) | NA | NA | Inactive | Plausible  (1006) | NA | NA |
| **DCF-10** |  | NA | Equivocal  (035) | NA | NA | Inactive | Plausible  (1006) | NA | NA |
| **DCF-11** |  | NA | Equivocal  (035) | NA | NA | Inactive | NA | Plausible  (116) | NA |
| **DCF-12** |  | NA | NA | NA | NA | Plausible  (327) | NA | Plausible  (116, 743) | NA |

Table S4*. In silico* toxicity assessment of IBU and its transformation products (TPs) using Derek Nexus (NA - „No alerts”; structural alerts codes are given in brackets: tertiary alcohol or ether (048); 1,2-ethyleneglycol or derivative (069); 2-arylacetic or 3-arylpropionic acid (620)).

| **IBU and its TPs** | **Chemical structure** | | **Hepatotoxicity**  **in mammal** | **Nephrotoxicity**  **in mammal** | **Neurotoxicity** | **Estrogenicity** | **Mutagenicity**  ***in vitro***  **in bacterium** | **Chromosome damage *in vitro* in mammal** | **Carcinogenicity** | **Teratogenicity** |
| --- | --- | --- | --- | --- | --- | --- | --- | --- | --- | --- |
| **IBU** |  | Plausible  (620) | | NA | NA | NA | Inactive | NA | NA | NA |
| **IBU-1** |  | Plausible  (620) | | NA | NA | NA | Inactive | NA | NA | NA |
| **IBU-2** |  | Plausible  (620) | | Equivocal  (048) | NA | NA | Inactive | NA | NA | NA |
| **IBU-3** |  | Plausible  (620) | | NA | NA | NA | Inactive | NA | NA | NA |
| **IBU-4** |  | Plausible  (620) | | Equivocal  (069) | NA | NA | Inactive | NA | NA | NA |
| **IBU-5a** |  | NA | | NA | NA | NA | Inactive | NA | NA | NA |
| **IBU-5b** |  | NA | | Equivocal  (048) | NA | NA | Inactive | NA | NA | NA |
| **IBU-5c** |  | NA | | NA | NA | NA | Inactive | NA | NA | NA |

Table S5. In silico toxicity assessment of KET and its transformation products (TPs) using Derek Nexus (NA - „No alerts”).

| **KET and its TPs** | **Chemical structure** | **Hepatotoxicity**  **in mammal** | **Nephrotoxicity**  **in mammal** | **Neurotoxicity** | **Estrogenicity** | **Mutagenicity**  ***in vitro***  **in bacterium** | **Chromosome damage *in vitro* in mammal** | **Carcinogenicity** | **Teratogenicity** |
| --- | --- | --- | --- | --- | --- | --- | --- | --- | --- |
| **KET** |  | Plausible  (620) | Plausible  (801) | NA | NA | Inactive | NA | Plausible  (707) | NA |
| **KET-1a** |  | Plausible  (620) | Plausible  (801) | NA | NA | Inactive | NA | Plausible  (707) | NA |
| **KET-1b** |  | Plausible  (620) | Plausible  (801) | NA | NA | Inactive | NA | Plausible  (707) | NA |
| **KET-1c** |  | Plausible  (620) | Plausible  (801) | NA | NA | Inactive | NA | Plausible  (707) | NA |
| **KET-1d** |  | Plausible  (620) | Plausible  (801) | NA | NA | Inactive | NA | Plausible  (707) | NA |
| **KET-1e** |  | Plausible  (620) | Plausible  (801) | NA | NA | Inactive | NA | Plausible  (707) | NA |
| **KET-1f** |  | NA | NA | NA | NA | Inactive | NA | Plausible  (707) | NA |
| **KET-2** |  | Plausible  (620) | Plausible  (801) | NA | NA | Inactive | NA | Plausible  (707) | NA |
| **KET-3** |  | Plausible  (620) | Plausible  (801) | NA | NA | Inactive | NA | Plausible  (707) | NA |
| **KET-4** |  | Plausible  (620) | Plausible  (801) | NA | NA | Inactive | NA | NA | NA |

**References**

1.	Marco-Urrea, E., Pérez-Trujillo, M., Cruz-Morató, C., Caminal, G. & Vicent, T. White-rot fungus-mediated degradation of the analgesic ketoprofen and identification of intermediates by HPLC-DAD-MS and NMR. *Chemosphere* **78**, 474–481 (2010).

2.	Choi, S. *et al.* A validated chiral LC–MS/MS method for the enantioselective determination of (S)-(+)- and (R)-(-)-ibuprofen in dog plasma: Its application to a pharmacokinetic study. *Pharmaceutics* **15**, (2023).

3.	Skordi, E., Wilson, I. D., Lindon, J. C. & Nicholson, J. K. Characterization and quantification of metabolites of racemic ketoprofen excreted in urine following oral administration to man by 1H-NMR spectroscopy, directly coupled HPLC-MS and HPLC-NMR, and circular dichroism. *Xenobiotica* **34**, 1075–1089 (2004).
